# Theoretical study of the effect of coordination environment on the activity of metal macrocyclic complexes as electrocatalysts for oxygen reduction

**DOI:** 10.1016/j.isci.2022.104557

**Published:** 2022-06-08

**Authors:** Ziqi Tian, Yuan Wang, Yanle Li, Ge Yao, Qiuju Zhang, Liang Chen

**Affiliations:** 1 Ningbo Institute of Materials Technology and Engineering, Chinese Academy of Sciences, Ningbo 315201, Zhejiang, China; 2 University of Chinese Academy of Sciences, 100049 Beijing, China; 3 Nano Science and Technology Institute, University of Science and Technology of China, Suzhou 215123, China; 4 School of Physics, Collaborative Innovation Center of Advanced Microstructures, and National Laboratory of Solid State Microstructures, Nanjing University, Nanjing 210093, China

**Keywords:** chemistry, catalysis, electrochemistry, computational chemistry

## Abstract

Transition metal macrocyclic complexes are appealing catalysts for electrochemical oxygen reduction reaction (ORR). Here, we perform first-principles calculations to gain a comprehensive understanding on the structure-property relationship of the metal macrocyclic complex systems. Various modifications of the complexes are considered, including centered metal, axial ligand, coordination atom, substituent, and macrocycles. Based on simulation, introduction of appropriate apical ligand can improve the performance of all the three metals, whereas replacement of nitrogen with oxygen or carbon as the coordination atoms may enhance the Ni-centered systems. The antiaromatic ring stabilizes the ∗OOH intermediate, whereas the macrocycle with reduced electron density inhibits the binding with oxygen. By regulating the coordination environment, the overpotential can be significantly reduced. This work may assist the rational design of ORR catalysts and is of great significance for the future development of oxygen reduction catalysts.

## Highlights

• Metal macrocyclic complexes are potential electrocatalysts for ORR
• An understanding on structure-property relationship is gained based on simulation
• Various modifications are considered to improve the performance

## Graphical abstract

**Figure:**
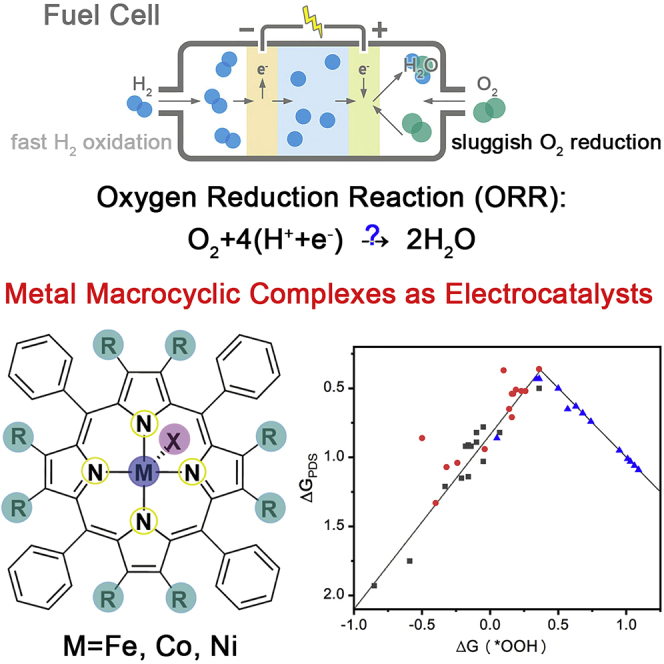

## Introduction

Hydrogen energy provides an attractive way to decarbonize many economic sectors, including transport and power generation (Brandon and Kurban, 2017; Nolan and Browne, 2020). Fuel cell systems play a key role in hydrogen energy technology (Dodds et al., 2015; Akal et al., 2020). The hydrogen-powered vehicles have been available in the market, whereas there are still many challenges that hampered its widespread commercialization (Jacobson et al., 2005; Wilberforce et al., 2017). One of the most urgent issues to be addressed is the development of efficient and economical cathode materials (Banham et al., 2015; Tahir et al., 2022). In most fuel cells, oxygen reduction reaction (ORR) takes place at the cathode (Xiao et al., 2021), in which oxygen is reduced to water via a four proton-electron transfer process:

O_2_+4e^−^+4H^+^→2H_2_O (1)

Because the generation of water requires transfer of multiple electrons, the reaction suffers from sluggish kinetics and high overpotential (Qin et al., 2021). Currently, platinum-based materials are the most commonly used commercial catalysts (Shao et al., 2018). But their large-scale application is limited by the scarcity and the high price of the noble metal. Designing high-performance catalysts using base metal is highly desired.

Metal macrocyclic complexes, such as heme and other metalloporphyrins, are pivotal components in enzyme systems that perform as reactive centers to catalyze biochemical redox reactions with extremely high activity (Li et al., 2021). Since cobalt phthalocyanine was employed as an ORR catalyst by Jasinski in 1964 (Jasinski, 1964), these complexes have attracted much attention in electrochemistry. Much effort has been devoted to promoting the catalytic activity of metal macrocyclic complexes (Liu et al., 2016; Zhang et al., 2017). Numerous systems containing metal macrocyclic complex subunits have been synthesized. Especially, the Fe-centered and Co-centered systems are widely studied as the promising electrocatalysts for ORR. For example, using Fe/Co-phthalocyanines as the functional subunits, Yang prepared two-dimensional conjugated aromatic networks via a one-step ball milling of the solid-phase synthesis (Yang et al., 2019). The materials display outstanding ORR mass activity, even beyond commercial Pt/C. Cichocka et al. report a series of Zr-based metal organic frameworks (MOFs) in which functional metalloporphyrins are grafted as the electrochemical active sites for ORR (Cichocka et al., 2020). The stable Zr-MOF platforms tailor the immobilization and packing of metal macrocyclic subunits, significantly enhancing ORR activity. Yue et al. fabricated two-dimensional covalent organic frameworks (CO-Fs) composed of metalloporphyrins and donor-acceptor dyads for electrochemical ORR (Yue et al., 2021). The Co-centered COF exhibits the best performance among their studied materials.

Moreover, derived from the metal macrocyclic complexes, a series of single atom catalysts (SACs), also known as M-N-C systems, have been investigated extensively and intensively in past ten years (Wang et al., 2018; Li et al., 2020a, 2020b; Peng et al., 2020; Zhao et al., 2021). As characterized by XANES and other spectrums, their active sites have many features in common with the corresponding metal macrocyclic complexes. These materials marry the advantages of enzyme and heterogeneous catalysts, exhibiting outstanding performance on electrocatalysis systems, including ORR. For example, Chen et al. made a highly reactive and stable Fe/N-doped porous carbon. The ORR performance outperforms the commercial Pt/C catalysts, with low overpotential, high kinetic current density, and outstanding stability (Chen et al., 2017). Experiments and simulations demonstrated that the isolated Fe-N4 sites were crucial to deliver the outstanding performance. Jiao et al. synthesized Fe-N-C SAC via pyrolysis of porphyrinic metal–organic frameworks (Jiao et al., 2018). The materials possess high content of single-atom Fe-N4 sites, hierarchical pores, oriented nano-channels and high conductivity, leading to ultimate ORR activity. Lin et al. employed an open framework platform with a large number of chelating ligands to prepare a series of SACs containing Fe-N5 site (Lin et al., 2019). By increasing the coordination number of the metal site, the interaction with the key intermediate is modulated. Excellent ORR activity with a half-wave potential of 0.89 V and high stability are achieved. On the other hand, the active site structures in the heterogeneous catalysts are quite difficult to be well-defined. The metal macrocyclic complexes can be seen as the model systems of the active sites, based on which one can understand the effect of the coordination environment on catalytic activity in depth.

Furthermore, theoretical investigations have also been performed to give insight into the catalytic mechanism and structure-property relationship. Seo et al. compared the electronic structures of ferrous phthalocyanine and its derivative that was modified with diphenylphenthioether substituent (Seo et al., 2014). The ORR activity could be well regulated by the incorporation of functional groups. The relative position of the metal *d*z2-orbital can be controlled by the incorporation of functional groups, leading to the tunable ORR activity. Ni et al. computationally investigated the relationship between the aromaticity/antiaromaticity of the macrocycles and the activity of transition metal centered complexes as ORR electrocatalysts (Ni et al., 2021). The antiaromatic macrocyclic ligand can enhance adsorption strength with oxygenated intermediate. Metal centers require matching macrocycles to improve ORR activity. Xu et al. systematically screened numerous metal macrocyclic complexes and graphene-based single-atom catalysts toward ORR and other electrocatalysis reactions, indicating that the activity is highly correlated with the chemical environment of the metal center, including coordination number and the electronegativity of the coordination atoms (Xu et al., 2018).

In this work, we take metalloporphyrins as the starting point to study the effects of different modifications on the performance of ORR electrocatalysis. Porphyrins are typical macrocyclic ligands in coordination chemistry, which can accommodate many transition metal ions to form macrocyclic complexes (Zhang and Warren, 2021). The extended aromatic structure of the macrocycle can support a range of oxidation states, stabilizing critical intermediates in the redox reactions. In addition, porphyrins provide a versatile platform for functionalization, thus fine-tuning of the intrinsic properties is available. Here, the catalytic pathways of a series of metalloporphyrins on ORR have been systematically studied by applying density functional theory (DFT) calculation. Several modifications are considered to modulate the performance, such as substituents, apical ligand, and elements coordinated with metal. Three more macrocycle ligands are also included further. The theoretical results show that Fe and Co complexes bind with oxygenated intermediates much more intensely than that containing Ni. By introducing proper apical ligand and modifying the coordination atoms and the macrocyclic structure, the formation energies of these key intermediates are tunable. On the other hand, the effect of substituent is not significant. In particular, in this work, the macrocyclic complexes are considered as the reaction centers in porous frameworks and functional motifs in SACs, thus the freestanding molecule models are utilized. In experiment, the macrocyclic complexes have also been supported on various substrates, such as carbon nanotubes (Zhang et al., 2009; Abarca et al., 2019; Govan et al., 2020; Loyola et al., 2021a, 2021b). Proper substrates may also play a critical role in the catalysis process as discussed in previous theoretical studies (Orellana, 2011, 2012).

## Results and discussion

**Figure 1:**
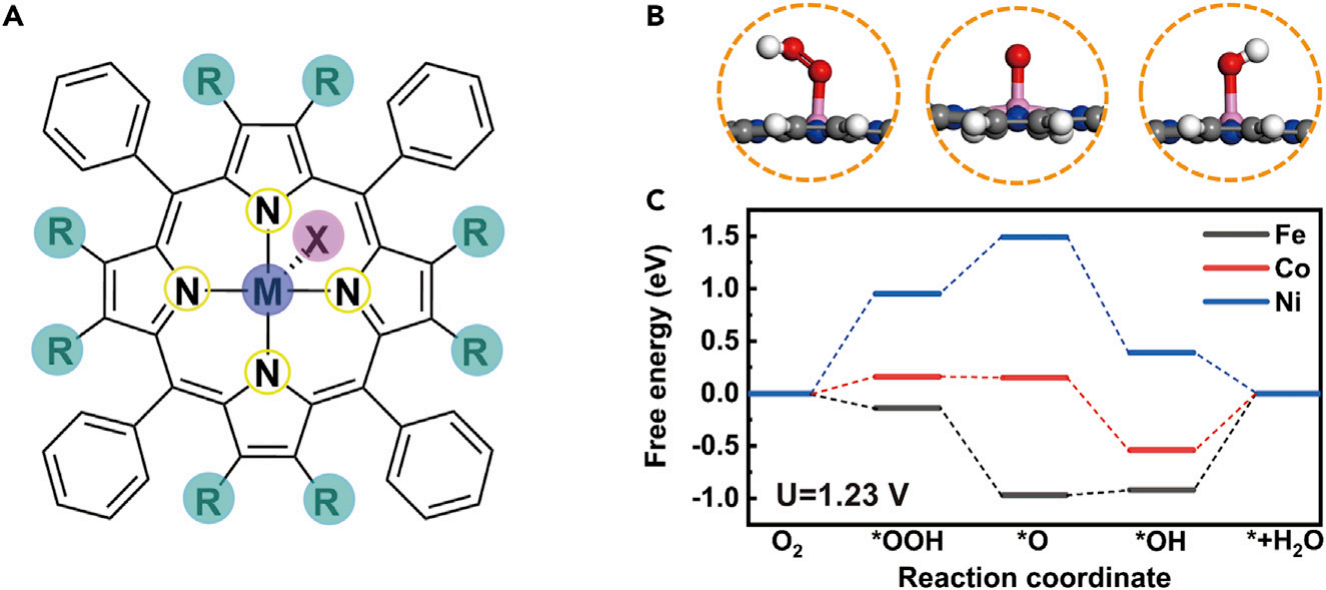
Schematic representation of the studied system and free energy diagrams of three typical catalysts (A) Structures of metal-centered tetraphenylporphyrin (PP). (B) Side views of three intermediates (∗OOH, ∗O and ∗OH) on the 4-electron ORR pathway. Color code: white, H; gray, C; blue, N; red, O; pink, metal. (C) Free energy diagrams of ORR processes catalyzed by Fe-, Co-, and Ni-PP complexes.

As shown in Figure 1A, tetraphenylporphyrin (PP) is first taken as the ligand, because it is a common functional motif in porous framework. The relatively large phenyl group can keep the planar structure. Three iron-group elements are studied as the center cations, i.e., Fe, Co, and Ni. Although the ORR mechanism is still an open question and several possible pathways have been proposed in literatures (Shi and Zhang, 2007; Sun et al., 2011; Ramaswamy and Mukerjee, 2011; He et al., 2012; Orellana, 2013), in this work our discussion is mainly based on the four-step path that goes through ∗OOH, ∗O and ∗OH intermediates (Figure 1B). The asterisk denotes the active metal site. We assume that the relative energies of the key intermediates are consistent with the energetics trend derived from this path even if the reaction follows other mechanisms. From the free energy profiles in Figure 1C, one can see that the potential-determining steps (PDSs) of Fe-PP and Co-PP systems are both the last step that ∗OH is reduced to water and desorbs from the catalyst. At an external potential of 1.23 V, the energy changes of the PDSs are 0.92 eV and 0.54 eV for Fe-centered and Co-centered complexes, respectively. To improve the activity of the whole ORR process, the interaction between metal and the oxygenated intermediate should be weakened. In the contrast, the PDS of Ni-PP-catalyzed path is the first step to form∗OOH with an energy change of 0.95 eV, namely that the binding to oxygen is too weak. The strength of the binding between metal and oxygenated species follows a trend as: Fe > Co > Ni. In experiment, Fe-containing and Co-containing systems have been widely studied as the candidates of electrocatalysts for ORR. Many investigations demonstrated that the ORR process on Fe-centered catalysts is limited by desorption of ∗OH, whereas the cobalt based materials bind oxygen weakly for efficient ORR (Zitolo et al., 2017; Pegis et al., 2019). Such inconsistency may be because of inaccurate description of the solvation effect and other systematic errors. But it is still worthwhile to investigate how the interaction can be regulated by modifying the coordination environment.

**Figure 2:**
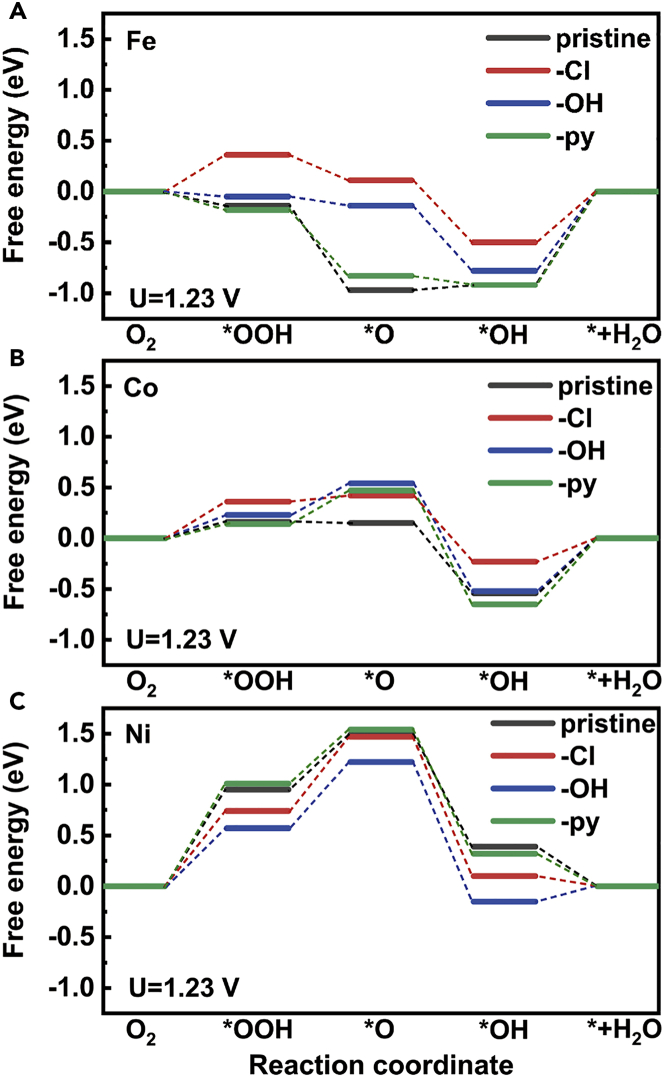
Free energy diagrams of ORR processes on three M-PPs with different axial ligands, i.e., Cl^−^, OH^−^ and pyridine (py) (A) Fe-centered complexes. (B) Co-centered complexes. (C) Ni-centered complexes.

To improve the ORR performance of metalloporphyrin, the adsorption strength of oxygenated intermediate is expected to be reasonably changed by modifying the coordination environment. In the three studied complexes, the coordination numbers of the centered cations are 4, indicating that the metal is unsaturated and able to coordinate with additional ligands in axial direction (the purple ligand in Figure 1A). In biochemistry, the apical ligand plays a critical role in the catalysis process (Liu et al., 2017). Herein, we study the influence of different apical ligands on the adsorption strength of oxygenated species on metalloporphyrins, including -Cl, -OH, and pyridine (py for short). The free energy profiles are depicted in Figure 2. Generally, the binding capacity of oxygenated intermediate is weakened after Fe-PP and Co-PP coordinating with the additional apical anion ligand. The less unsaturated Fe and Co centers exhibit weakened affinity to the oxygenated species. For example, the formation energies of ∗OOH species increase by 0.50 and 0.20 eV on Cl-coordinated Fe- and Co-PP, respectively. Meanwhile, the energy changes of the PDSs, namely desorption of ∗OH, decrease to 0.50 and 0.36 eV on Fe- and Co-complexes, related to improved oxygen reduction performance. In experiment, it has been also reported that the ultimate ORR performance is achieved by constructing a single atom catalyst with coordination number of five (Han et al., 2018; Li et al., 2022). In contrast, Ni^2+^ cation possesses *d*^8^ electron configuration. The crystal field splitting results in the unoccupied *d*_*x2-y2*_ orbital much higher than the other four occupied *d*-orbitals. The electron transfer to the unoccupied orbital is energetically unfavorable. After introducing anion as the axial ligand, the center cation becomes a *d*^*7*^ configuration that can accept one more electron and ligand to form a stable state. As a result, the binding between Ni and oxygenated species is strengthened. Free energy changes of ∗OOH formation decreases from 0.95 eV to 0.74 and 0.65 eV with additional -Cl and –OH ligands, respectively. Therefore, the overall oxygen reduction performance is improved by introducing additional ligands as well. Previously, Tasca et al. (Cao et al., 2013; Riquelme et al., 2018; Viera et al., 2020; Loyola et al., 2021a, 2021b; Govan et al., 2021; Oyarzun et al., 2021) systematically studied similar Fe-centered and Co-centered complexes with axial ligands by combining experiments and theoretical calculation. They also demonstrated that the additional coordination promotes the overall ORR performance. Yet, they supposed that the additional pyridine-like ligands improve catalytic activity mainly by enhancing the binding between metal and O_2_ molecules. Therefore, the whole mechanism is still worth discussing in future. In particular, the kinetic process of the reaction and the solvent effect should be included in in-depth mechanistic studies.

**Figure 3:**
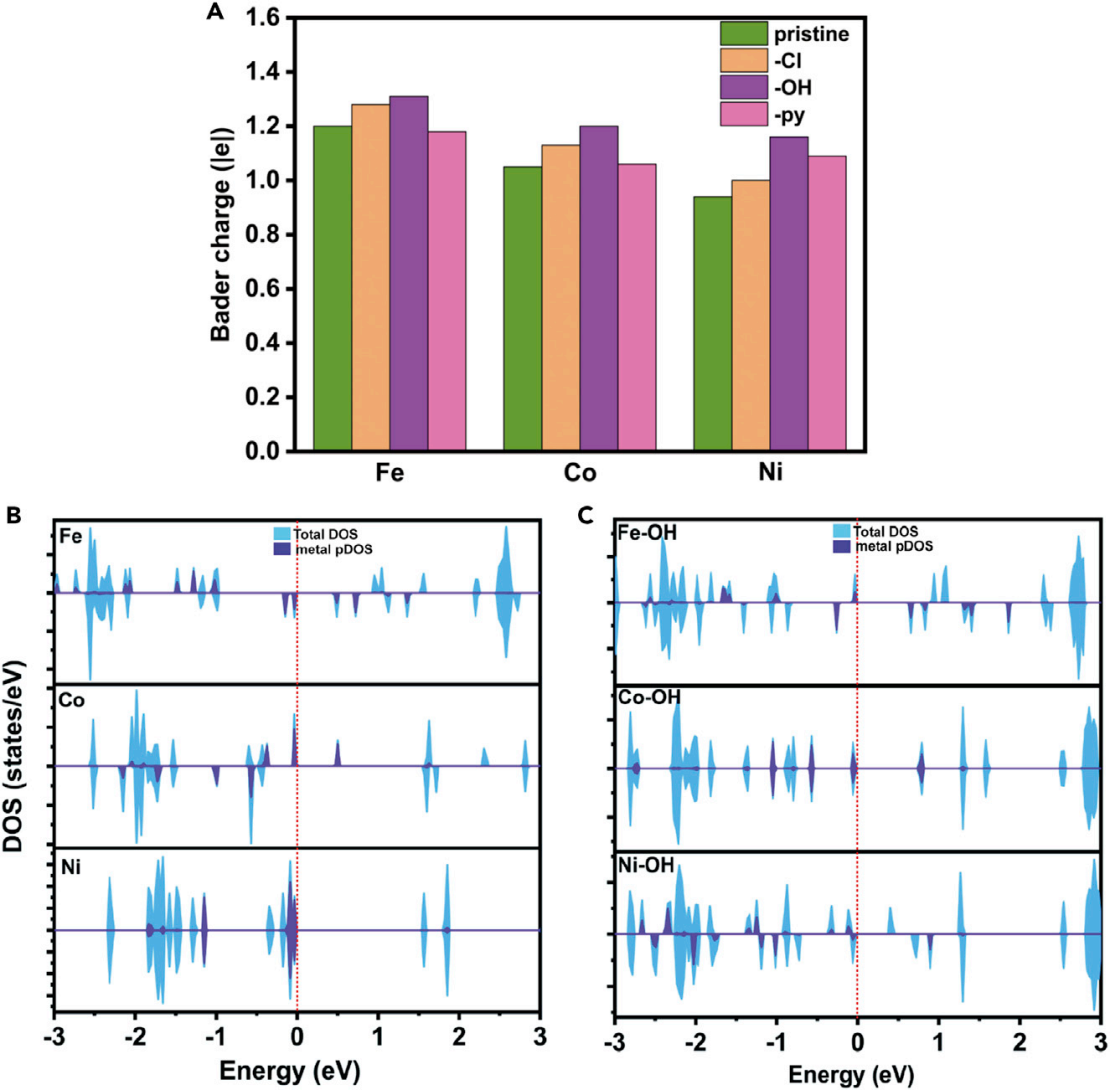
Property analysis of PP-coordinated complexes without/with additional ligands (A) The atomic charges of metal centers. (B) DOSs of Fe-, Co-, and Ni-PP systems. (C) DOSs of Fe-, Co-, and Ni-PP systems with axial -OH ligands.

Moreover, the atomic charge and density of state (DOS) are analyzed to give more insight of the additional ligand effect. As shown in Figure 3A, as coordinating with the axial anion ligands, the partial charges of Fe and Co become more positive, indicating electron depletion of the metal center. Fewer valence electrons lower the Fermi level, making it more difficult to form new bonds with oxygen. The DOS in Figures 3B and 3C also illustrates that the energy of the unoccupied orbitals rises and the band gaps increase after Fe and Co bind to -OH. On the other hand, although the binding to the axial anion also results in a more positive partial charge on Ni cation, the electron structure changes as well. The orbital degeneracy is removed and the band gap is apparently reduced, corresponding to the easier formation of new Ni-O bonds.

Introducing substituent is another common strategy to modulate the properties of the macrocycle (Wannakao et al., 2017; Abarca et al., 2019; Govan et al., 2020; Oyarzun et al., 2021). Here, we considered four substituents on the pyrrole subunits, i.e., -F, -Cl, -Br, and -CH_3_ (the green groups in Figure 1A). As in Figure S1, these free energy diagrams of ORR processes represent that the substituents have little impact on the binding to the oxygenated intermediates. The energy change of each step is within 0.2 eV. Thus the PDS on each metal remains unchanged, namely that the interaction between oxygen and Fe or Co is too strong while that between oxygen and Ni is too weak. For Fe and Co centered systems, the best performance can be achieved when the substituents are -Cl and -CH_3_, respectively, with energy changes of PDSs as 0.82 and 0.37 eV at external potential of 1.23 eV. For Ni centered systems, the substituents cannot improve ORR performance effectively. Although it is difficult to obtain a general rule from the computational results, one can finely modulate the interactions between metal and oxygenated species by introducing suitable substituents. To tailor the binding strength more effectively, other modification is necessary.

**Figure 4:**
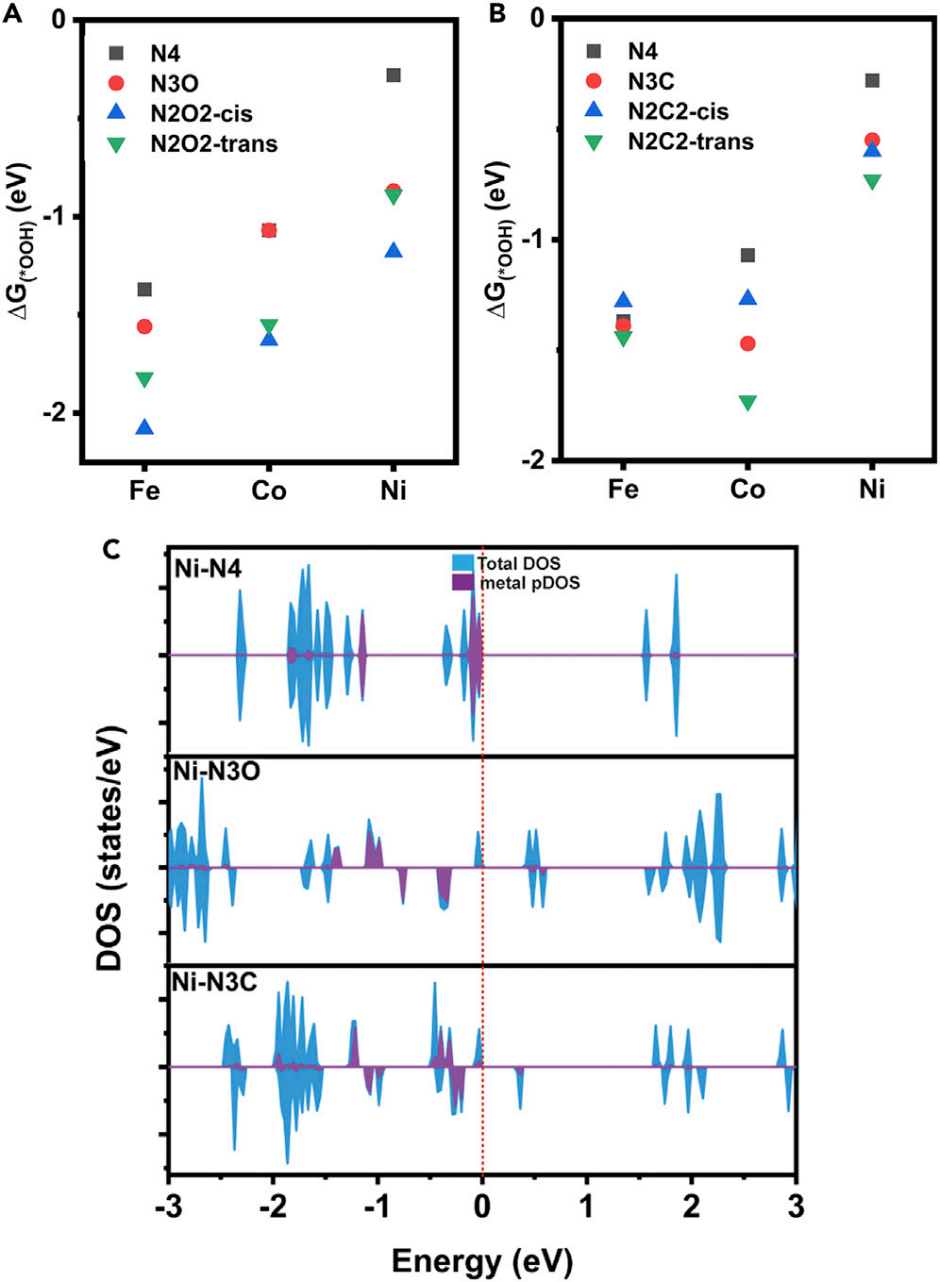
Investigation on the heteroatom substituted PPs in which one or two nitrogen atoms are replaced (A) The relationship between ΔG(∗OOH) and oxygen-substituted PPs. (B) The relationship between ΔG(∗OOH) and carbon-substituted PPs. (C) The DOSs of three Ni-centered macrocyclic complexes.

The porphyrin molecule is composed of four pyrrole subunits, which may be replaced by other heterocycles, such as furan or cyclopentadienyl group. In many prepared SACs, metal centers can bind with carbon and oxygen beyond nitrogen. Thus one or two nitrogen atoms that coordinate with metal (the nitrogen atoms in yellow circle in Figure 1A) are replaced by oxygen or carbon (Li et al., 2020a, 2020b; Tang et al., 2021). We regard six C/O-replaced PP molecules, marked as N3O, N2O2-cis, N2O2-trans, N3C, N2C2-cis, and N2C2-trans. These labels represent the atoms coordinating with metal. There are two configurations for the PP molecule in which two nitrogen atoms are replaced by oxygen/carbon atoms, i.e., the cis and the trans configurations. The free energy profiles are shown in Figure S2, and ΔG(∗OOH)s are analyzed to evaluate the binding strengths between metal and oxygenated species. As plotted in Figures 4A and 4B, the replacement of nitrogen by either oxygen or carbon enhances the interaction of the metal center with the oxygenated intermediate. Therefore, ORR is suppressed on Fe and Co centers; whereas the Ni center catalyzes the reaction efficiently by replacing nitrogen atoms with oxygen or carbon. From the DOSs of three Ni-centered complexes in Figure 4C, it can be found that the replacement of heteroatom completely changes the electronic structure of the complex. The highest occupied orbital is mainly composed of the macrocycle’s orbital. The band gap is consequently narrowed, facilitating the reaction with oxygen.

Beyond the porphyrin macrocycles, other expanded porphyrins have been synthesized (Jasat and Dolphin, 1997; Saito and Osuka, 2011). We studied three more porphyrin-like macrocyclic molecules as ligands, labeled as L1, L2, and L3 in Figure 4A, respectively. As shown in Figures 4B–4D, L1 (tetrabenzoporphyrin) possesses the same coordination configuration and macrocyclic structure as PP, thus the M-L1 systems exhibit very similar performance to that of M−PP systems, in consistent with aforementioned conclusion that the substituents have little impact on the interaction of centered cation to oxygenated groups. Although if the macrocycle L2 (corrole) is taken as the ligand, the affinities of all three cations to ∗OOH are enhanced apparently. As a result, performance of Ni-L2 on ORR is improved but that of Fe-L2 and Co-L2 are inhibited. ΔG(∗OOH) of Ni-L2 system decreases from 0.95 eV to 0.71 eV at an external potential of 1.23 V. Previous study demonstrated that the complexes with antiaromatic macrocycles significantly enhance adsorption strengths, mainly because of the various redox activities of macrocyclic ligands with different aromaticities (Ni et al., 2021). The antiaromatic ligand is more likely to accept electrons to become a stable state. The redox activity of ligands further affects the activity of metal via d-π conjugation. In this case, L2 is an antiaromatic macrocycle, thus our results are in consistent with Ni’s conclusion. On the other hand, four α carbon atoms in porphyrin are replaced by nitrogen atoms to generate L3 (5,10,15,20-tetraazaporphyrin), keeping the number of π electrons as 18. Phthalocyanine—a widely studied complex—can be seen as a derivative of L3. L3 coordination with metal results in the weakened interaction with ∗OOH and ∗OH. For instance, at a potential of 1.23 eV, the energy change of the desorption step (∗OH→H_2_O) decreases from 0.92 eV to 0.73 eV on Fe-centered macrocycle, and from 0.54 eV to 0.42 eV on Co-centered macrocycle, corresponding to the promoted ORR performance. In comparison, the electron structures of PP and L3 are quite similar. Although the unsaturated α nitrogen atoms insert into the large conjugate system, reducing electron density of the macrocycle and withdrawing electrons from the center cation. According to Bader charge analysis, the partial charges of the three cations all become more positive after replacing ligand PP with L3, i.e., from +1.20|e| to +1.21|e| for Fe, from +1.05|e| to +1.12|e| for Co and from +0.94|e| to +1.02|e| for Ni. The low electron density of the reaction site makes it difficult for oxygen to gain electrons and inhibits the ORR process.

We summarize the energy changes of PDSs (ΔG_PDS_) in Figure 6. By modifying the coordination environment, the activities of the metal macrocyclic systems are significantly altered. ΔG_PDS_ can be reduced by approximately 0.4 eV. In our studied systems, Co-PP-Cl possesses the best calculated performance that ΔG_PDS_ is 0.36 eV. Even for these Ni-centered complexes that are not normally considered as effective ORR catalysts, relatively acceptable performance may also be achieved by changing the coordination elements, such as Ni-N3O and Ni-N2O2-trans systems with the same ΔG_PDS_ as 0.43 eV.

**Figure 5:**
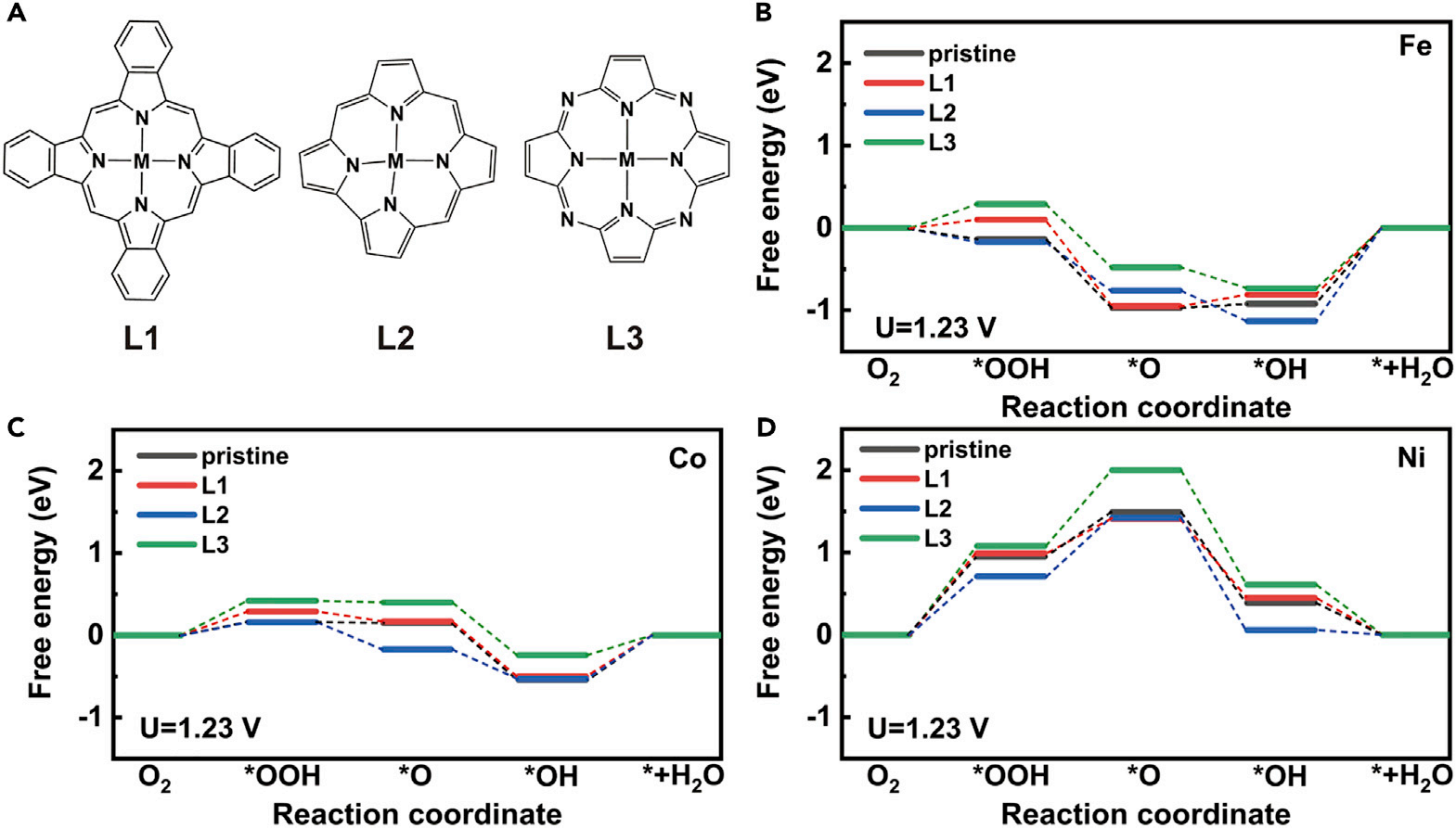
Study on other macrocyclic ligands (A) Structures of three studied macrocyclic ligands. (B) Free energy profiles of ORR processes catalyzed by Fe-centered macrocycles. (C) Free energy profiles of ORR processes catalyzed by Co-centered macrocycles. (D) Free energy profiles of ORR processes catalyzed by Ni-centered macrocycles.

**Figure 6:**
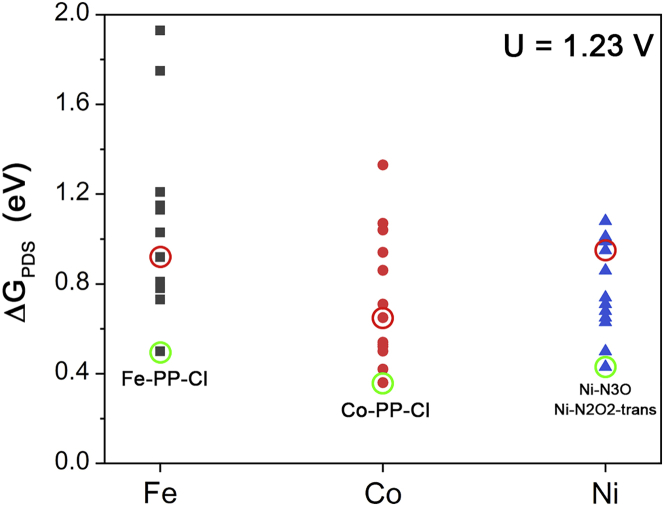
Summary of ΔG_PDS_’s of all our simulated systems

**Figure 7:**
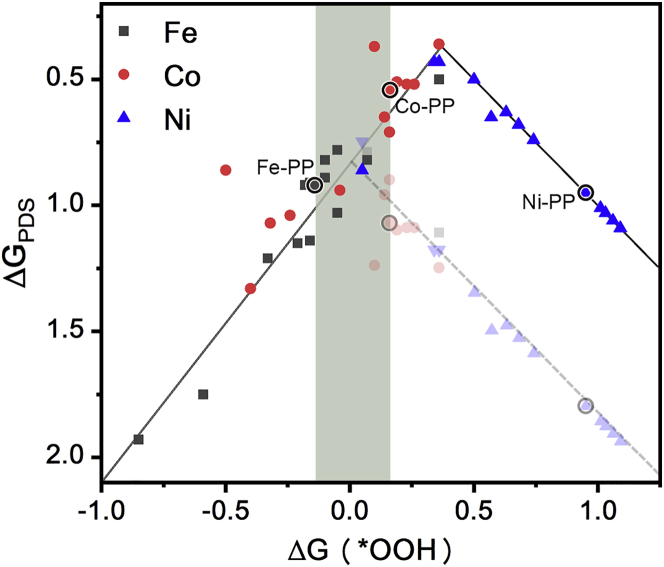
The volcano correlation of calculated ΔG(∗OOH) versus ΔG_PDS_ According to reported experiments and linear scaling relationship, the points with more positive ΔG(∗OOH)’s than the average value of Fe-PP and Co-PP are approximately moved down in gray. The area with ΔG(∗OOH) between Fe-PP and Co-PP is marked in shadow.

Finally, we can compare our calculation with existing experiments. Volcano or linear correlations between various descriptors and the ORR activity have been widely reported, such as binding energy of oxygen molecule (and other oxygen-containing species), M^III^/M^II^ cation redox potential, number of *d-*electrons, and the intermolecular hardness (Zagal and Koper, 2016; Kumar et al., 2020; Loyola et al., 2021a, 2021b). Here we plot the relationship of ΔG(∗OOH) versus ΔG_PDS_ in Figure 7, which also exhibits typical volcano correlation. In this volcano plot, one can see that Fe-centered and Co-centered species are on the left side corresponding to too strong metal-oxygen binding, whereas Ni-centered species on the right side corresponding to weak binding. If the binding between metal and oxygen is too weak, the M−O bond is more likely to break than the O-O bond, leading to the generation of H_2_O_2_. Numerous experiments have illustrated that Fe-O binding is too strong for the regeneration of catalyst (Cao et al., 2013; Sun, 2019; Loyola et al., 2021a, 2021b), whereas Co- and Ni-containing systems promote the H_2_O_2_ production (Riquelme, 2018; Jia and Yao, 2020; Wang et al., 2022). Therefore, our calculation may systematically overestimate the binding strength between oxygen and metal, probably because of the deviation of calculation method and inaccurate consideration of solvent. The peak of the volcano plot should be between the points corresponding to Fe-PP and Co-PP. We roughly revise the volcano plot by moving the right part down, as shown in the gray points and line in Figure 7. Then one can still infer that these points between or close to Fe-PP and Co-PP (in the shadow area of Figure 7) are promising electrocatalysts for ORR, including not only the synthesized systems (Fe-PP-Py (Cao et al., 2013; Loyola et al., 2021a, 2021b), Fe-PP-OH (Wang et al., 2019) Co-PP-Py (Riquelme et al., 2018; Viera et al., 2020; Govan et al., 2021) and substituted Fe-PP (Abarca et al., 2019; Govan et al., 2020; Oyarzun et al., 2021), but also some hypothesized models, such as Co-N3O and Ni-N2O2-cis. We expect experimental chemists to prepare these structures and test the performance in the future.

### Conclusion

In this work, a series of iron-group metal centered macrocyclic complexes have been systematically studied as potential electrocatalysts on ORR by using density functional theory (DFT). The binding between metal and oxygenated intermediate can be effectively regulated by modifying the coordination environments. By designing suitable ligands, optimal performance on ORR can be achieved. Specifically, introducing the anion as the axial ligand and replacing α carbon of macrocycle with nitrogen reduce the electrons on Fe and Co center, lowering the energy change of ∗OH desorption and thus improving ORR activity. On the other hand, replacing coordination sites as oxygen or carbon and changing the macrocycle to a nonaromatic system may increase the electrons on Ni center, enhancing the affinity to oxygenated intermediates and ORR performance as well. This investigation not only provides guidance for the design of novel materials that contain porphyrin-like subunits as reaction centers for electrocatalysis ORR but also points to design directions for the construction of high-performance heterogeneous single atom catalysts.

### Limitations of the study

The coupling of various features should be further studied in future. Solvation effect was roughly considered in this work, thus needs to be included in the in-depth study. The detailed reaction mechanism for the porphyrin system is still an open question. In most experiments, the binding between oxygenated species and the Co center is too weak for 4e-ORR to take place, whereas our results may overestimate the binding. Thus the simulation method could be further revised.

## STAR★Methods

### Key resources table

**Table:** 

REAGENT or RESOURCE	SOURCE	IDENTIFIER
**Software**		

VASP 5.4.4	Hafner (2008)	https://www.vasp.at
Adobe Photoshop	CC2018	https://www.adobe.com/
VESTA	Momma and Izumi (2011)	http://jp-minerals.org/vesta/
Materials Studio	BIOVIA, Dassault Systèmes	https://www.3ds.com/products-services/biovia/products/molecular-modeling-simulation/biovia-materials-studio/

### Resource availability

#### Lead contact

Further information and requests for resources should be directed to and will be fulfilled by the lead contact, Liang Chen (chenliang@nimte.ac.cn).

#### Materials availability

This study did not generate new unique reagents.

### Experimental model and subject details

(Omitted) Our study does not use experimental models typical in the life sciences.

### Method details

Spin polarized DFT calculation was performed by using the Vienna Ab initio Simulation Package (VASP) software package (Kresse and Furthmuller, 1996). PBE functional was employed with PAW method to describe the interaction between ions and electrons (Blochl, 1994; Kresse and Joubert, 1999). The cutoff energy of the plane wave was set to be 450 eV. To consider the solvent effect, the adsorption energies of ∗OH and ∗OOH species were subtracted by 0.50 and 0.25 eV, respectively, as suggested in literature (Rossmeisl et al., 2005). Since the isolated metal macrocyclic complexes are taken as the model systems, only the Γ-point is sampled. A vacuum layer of 15 Å in each direction was used to avoid the interaction between neighboring images under periodic boundary condition. All the structures have been fully relaxed. The optimized coordinations are attached as Data S1.zip in the supplement materials. The convergence criterion of the total energy and force was set to be 10^−4^ eV and 0.03 eV/Å, respectively.

The four-electron ORR pathway goes through the four elementary steps as (Rossmeisl et al., 2005; Fang and Liu, 2010):

∗+O_2_ (g) + H^+^ + e^−^→∗OOH

∗OOH + H^+^ + e^−^→∗O + H_2_O

∗O + H^+^ + e^−^→∗OH

∗OH + H^+^ + e^−^→∗+H_2_O

Based on the computational hydrogen electrode (CHE) model, the Gibbs free energy change (ΔG) is calculated as:

ΔG = ΔE + ΔE_ZPE_ - TS + ΔG_U_

where ΔE is the internal energy change directly calculated from DFT; ΔE_ZPE_ and TS refer to the change of zero-point energy and entropy for ∗OOH, ∗O, and ∗OH intermediates (Rossmeisl et al., 2007). Vibrational frequency calculations were carried out to obtain the zero-point energy and entropy. ΔG_U_ is deduced from –neU, where n is the number of transferred electrons and U is the external electrode potential vs. reversible hydrogen electrode (RHE) (Norskov et al., 2004; Hansen et al., 2008). In the following discussion, an external potential of 1.23 V is considered.

### Quantification and statistical analysis

(Omitted) Our study does not include quantification or statistical analysis.

## Data Availability

All the optimized structures of the metal macrocyclic complexes have been uploaded as Data S1.zip. Related to STAR Methods. Other data reported in this paper will be shared by the lead contact upon request. This paper does not report original code. Any additional information required to reanalyze the data reported in this paper is available from the lead contact upon request.

## Supplemental information

Document S1. Figures S1–S5 and Tables S1 and S2

Data S1. CONTCAR files of all the studied macrocyclic complexes
