# Supplementary material for: Theoretical study of the effect of coordination environment on the activity of metal macrocyclic complexes as electrocatalysts for oxygen reduction
Source: iScience. 2022 Jun 8;25(7):104557. doi: 10.1016/j.isci.2022.104557 (PMC9234223; doi:10.1016/j.isci.2022.104557)
Supplement: Document S1. Figures S1–S5 and Tables S1 and S2 [file mmc1.pdf]

**iScience, Volume *25***

## Supplemental information

# Theoretical study of the effect of coordination environment on the activity of metal macrocyclic complexes as electrocatalysts for oxygen reduction

**Ziqi Tian, Yuan Wang, Yanle Li, Ge Yao, Qiuju Zhang, and Liang Chen**

## This Supporting Information contains:

**Figure S1**. Free energy diagrams of oxygen reduction of Fe-PP, Co-PP and Ni-PP by using VASPsol. Related to STAR methods.

**Figure S2**. Free energy diagrams of oxygen reduction of M-PPs with various axial ligands by using VASPsol. Related to STAR methods.

**Figure S3**. Free energy diagrams of oxygen reduction of M-PPs with different substituents. Related to Figure 4.

**Figure S4**. Free energy diagrams of oxygen reduction of oxygen replaced PP systems. Related to Figure 4.

**Figure S5**. Free energy diagrams of oxygen reduction of carbon replaced PP systems. Related to Figure 4.

**Table S1.** The simulated states of all the reported metal complexes. Related to STAR methods.

**Table S2**. Energies of typical species in various simulation boxes. Related to STAR methods.

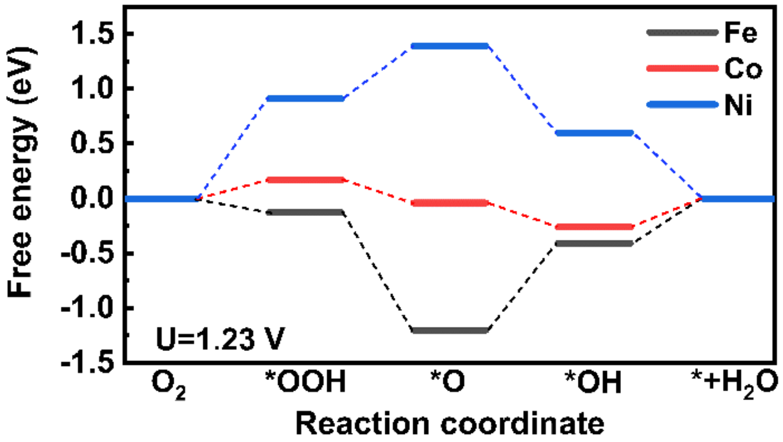


**Figure S1**. Free energy diagrams of oxygen reduction of Fe-PP, Co-PP and Ni-PP by using VASPsol to consider the solvent effect. Related to STAR methods.

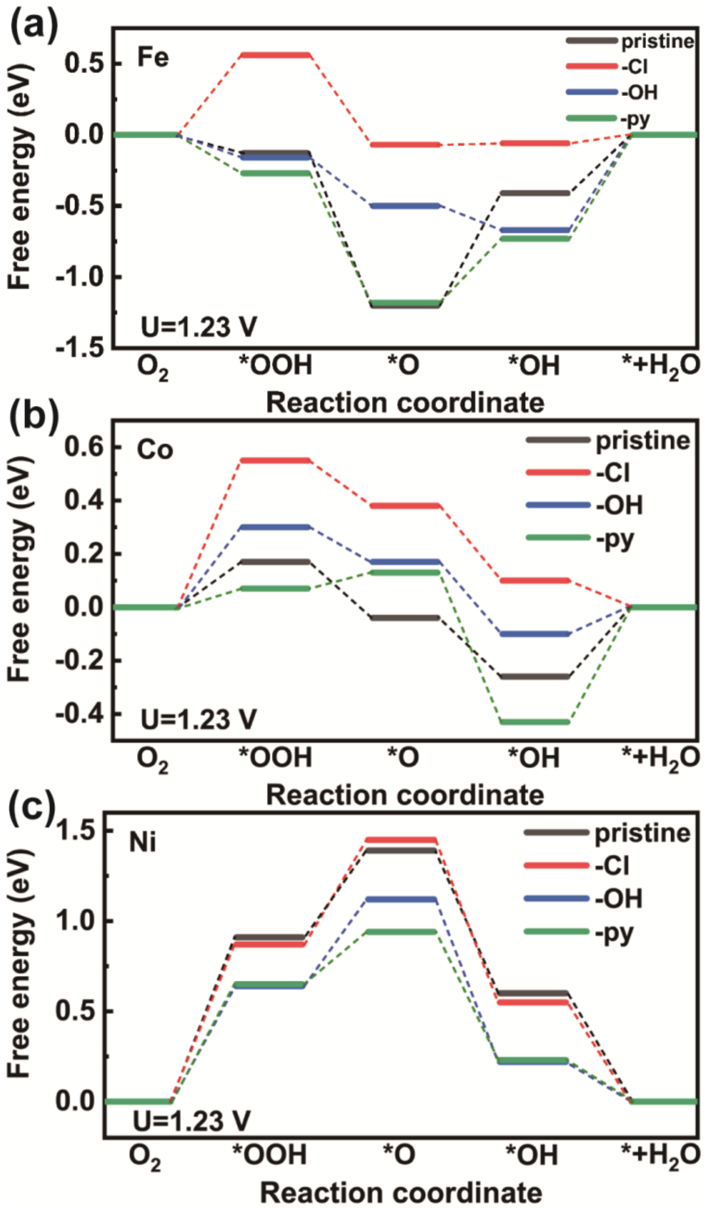


**Figure S2**. Free energy diagrams of oxygen reduction on (a) Fe-PPs, (b) Co-PPs and (c) Ni-PPs with various axial ligands by using VASPsol to consider the solvent effect. Related to STAR methods.

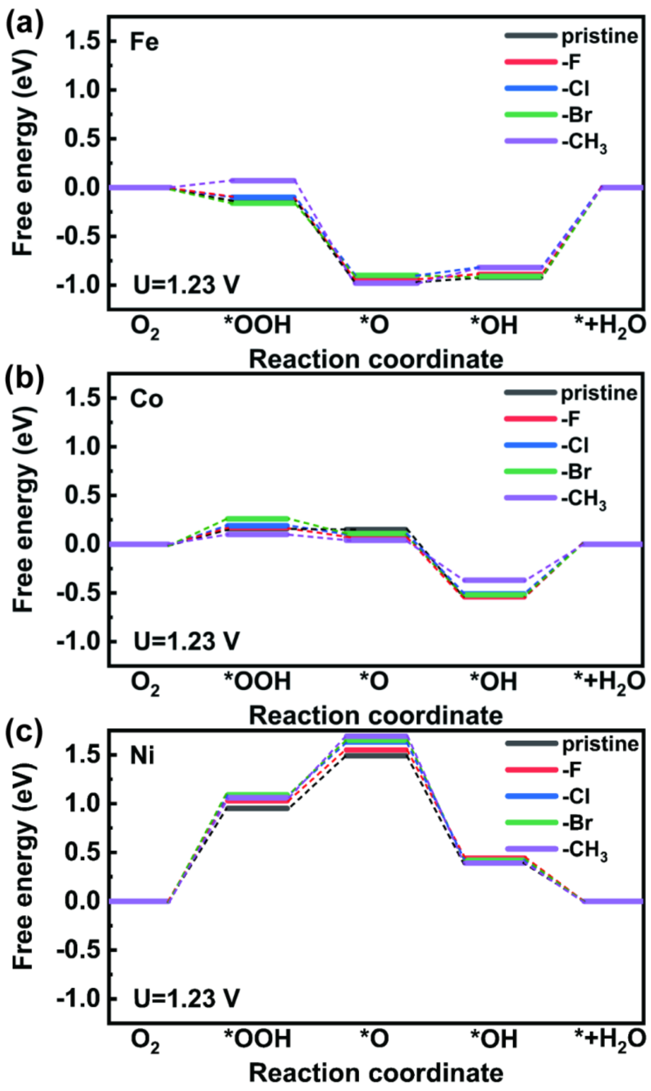


**Figure S3**. Free energy diagrams of oxygen reduction of (a) Fe-PPs (b) Co-PPs and (c) Ni-PPs with different substituents. Related to Figure 4.

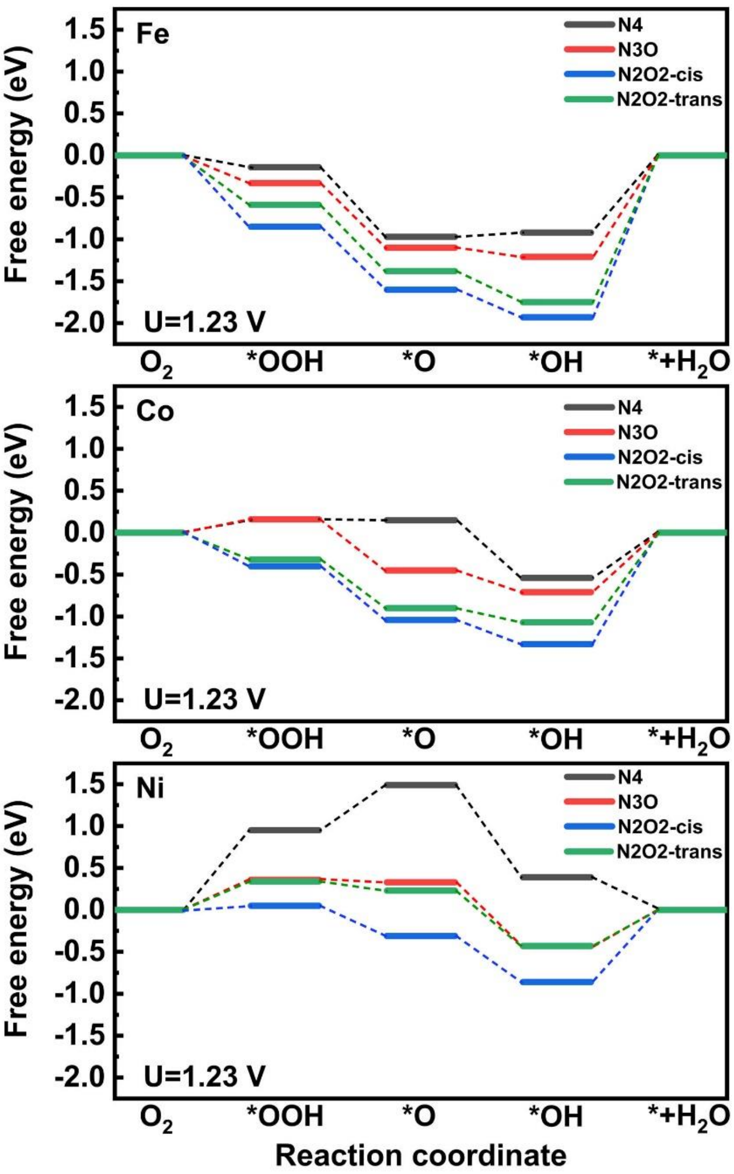


**Figure S4**. Free energy diagrams of oxygen reduction of oxygen replaced PP systems that coordinate with (a) Fe, (b) Co and (c) Ni. Related to Figure 4.

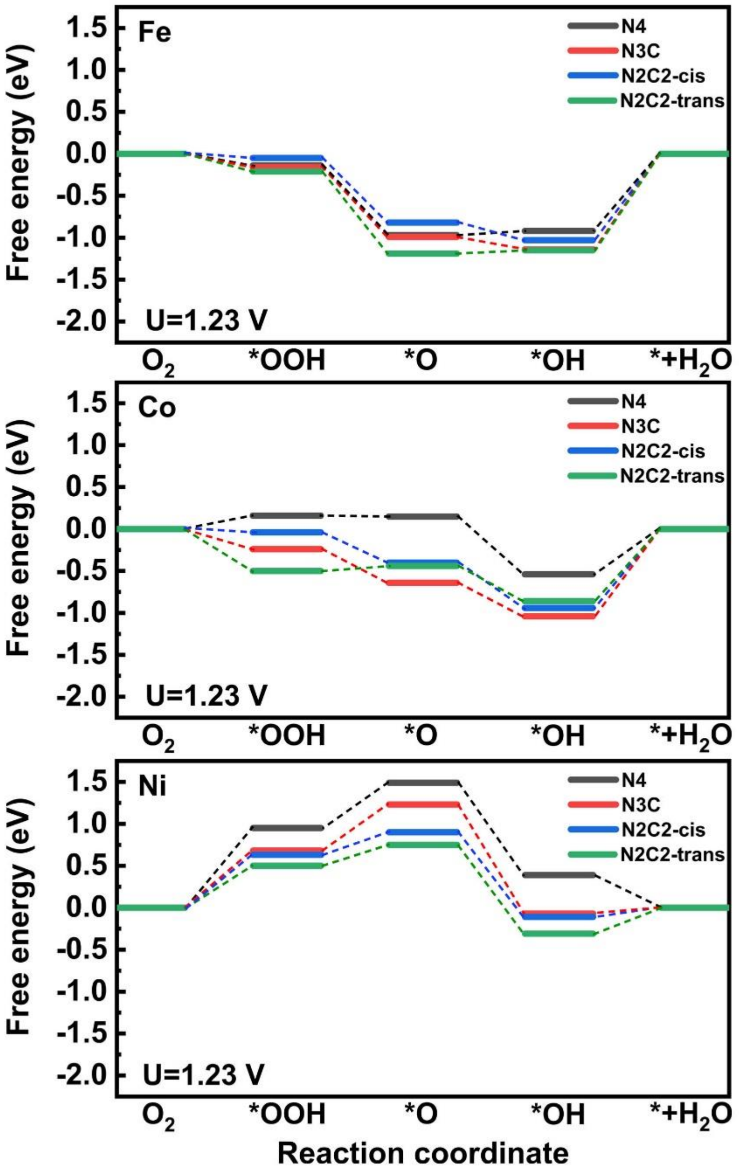


**Figure S5**. Free energy diagrams of oxygen reduction of carbon replaced PP systems that coordinate with (a) Fe, (b) Co and (c) Ni. Related to Figure 4.

**Table S1.** The simulated states of all the reported metal complexes. Related to STAR methods.

| | Magnetic moment (μB) | Magnetic moment (μB) | Magnetic moment (μB) |
|---|---|---|---|
| M= | Fe | Co | Ni |
| Coordination environment | | | |
| Pristine PP | 2.004 | 1.040 | 0.000 |
| With axial liagnd | | | |
| -Cl | 2.444 | 0.000 | 0.490 |
| -OH | 2.438 | 0.000 | 0.499 |
| -py | 0.000 | 0.967 | 0.000 |
| Substituents on pyrrole rings | | | |
| -Br | 1.969 | 0.995 | 0.000 |
| -CH3 | 1.979 | 1.005 | 0.000 |
| -Cl | 1.970 | 1.007 | 0.000 |
| -F | 1.981 | 1.034 | 0.000 |
| Replacements of the coordination atoms | | | |
| -N3O | 1.631 | 0.000 | 1.010 |
| -N2O2-cis | 3.304 | 2.121 | 1.056 |
| -N2O2-trans | 3.294 | 2.117 | 1.034 |
| Other macrocycles | | | |
| -L1 | 1.984 | 1.037 | 0.000 |
| -L2 | 2.414 | 1.355 | 0.217 |
| -L3 | 2.018 | 1.056 | 0.000 |

**Table S2**. Energies of typical species in various simulation boxes. Related to STAR methods.

| Box length | Calculaed energy / eV | | |
|---|---|---|---|
| | FePP | CoPP | NiPP |
| 20 Å | -539.490 | -538.153 | -536.903 |
| 25 Å | -539.495 | -538.153 | -536.901 |
| 30 Å | -539.493 | -538.154 | -536.906 |
